# Ion channels expression and function are strongly modified in solid tumors and vascular malformations

**DOI:** 10.1186/s12967-016-1038-y

**Published:** 2016-10-04

**Authors:** Antonella Biasiotta, Daniela D’Arcangelo, Francesca Passarelli, Ezio Maria Nicodemi, Antonio Facchiano

**Affiliations:** 1 Department of Neurology and Psychiatry, Sapienza University, Rome, Italy; 2 Istituto Dermopatico dell’Immacolata, IDI-IRCCS, Fondazione Luigi Maria Monti, via Monti di Creta 104, 00167 Rome, Italy

**Keywords:** Cancer, Ion-channels, Autonomic nervous system, Sympathetic skin response, SSR, Flat port-wine stains

## Abstract

**Background:**

Several cellular functions relate to ion-channels activity. Physiologically relevant chains of events leading to angiogenesis, cell cycle and different forms of cell death, require transmembrane voltage control. We hypothesized that the unordered angiogenesis occurring in solid cancers and vascular malformations might associate, at least in part, to ion-transport alteration.

**Methods:**

The expression level of several ion-channels was analyzed in human solid tumor biopsies. Expression of 90 genes coding for ion-channels related proteins was investigated within the Oncomine database, in 25 independent patients-datasets referring to five histologically-different solid tumors (namely, bladder cancer, glioblastoma, melanoma, breast invasive-ductal cancer, lung carcinoma), in a total of 3673 patients (674 control-samples and 2999 cancer-samples). Furthermore, the ion-channel activity was directly assessed by measuring in vivo the electrical sympathetic skin responses (SSR) on the skin of 14 patients affected by the flat port-wine stains vascular malformation, i.e., a non-tumor vascular malformation clinical model.

**Results:**

Several ion-channels showed significantly increased expression in tumors (p < 0.0005); nine genes (namely, CACNA1D, FXYD3, FXYD5, HTR3A, KCNE3, KCNE4, KCNN4, CLIC1, TRPM3) showed such significant modification in at least half of datasets investigated for each cancer type. Moreover, in vivo analyses in flat port-wine stains patients showed a significantly reduced SSR in the affected skin as compared to the contralateral healthy skin (p < 0.05), in both latency and amplitude measurements.

**Conclusions:**

All together these data identify ion-channel genes showing significantly modified expression in different tumors and cancer-vessels, and indicate a relevant electrophysiological alteration in human vascular malformations. Such data suggest a possible role and a potential diagnostic application of the ion–electron transport in vascular disorders underlying tumor neo-angiogenesis and vascular malformations.

## Background

Several key cellular functions are related to transmembrane potentials and lie under the control of ion channels, pumps and gap junction complexes. Controlling transmembrane voltage represents a fundamental process in many physiologically relevant steps, including cell cycle progression [1] and different forms of cell death [2, 3]. Over expression or increased activity of ion channels has been demonstrated as a response to mitogens exposure [4–6]. Several studies show a direct link between the transmembrane ion flow and carcinogenesis [7, 8]. However, as pointed out [9], the role membrane potential plays in the pathogenesis of several disorders, including cancer, is still not well understood. Plasma membrane de-polarization has a pivotal role at different stages of cell cycle progression and in various cell types [9]. Namely, endothelial cells hyper-polarization has been shown to contribute to cell division arrest [10], and channels are known to control migratory cellular properties in wound healing [11]. Further, Ca^2+^, K^+^ and Cl^−^ channels are essential regulators of cell proliferation and cancer development [12–16]. As recently further demonstrated, several ion-channels are directly involved in controlling tumor—[17] as well as non-tumor angiogenesis [18]. Expression and activity of TRPV4 channel have been found suppressed in tumor endothelium [19], and its activation has been found to normalize tumor vessels [20]; inhibiting Cl^−^ channel activity has anti-angiogenesis and anti-glioma properties [21]; finally, human voltage-dependent K^+^ channel has been found associated with cancer aggressiveness and angiogenesis [22]. Therefore, ion-channels play a fundamental role in cancer progression as well as in angiogenesis.

Flat port-wine stains are non-tumor malformations of the skin capillaries [23]. Cutaneous capillary malformations are usually isolated finding. However, they may occasionally coexist with cerebral or ocular vascular malformations, constituting the rare sporadic neurocutaneous Sturge-Weber syndrome (SWS) affecting the cephalic microvasculature, or may represent signs of more aggressive vascular malformations or even vascular tumors.

The sympathetic skin response (SSR) is an alteration in skin electrical potential evoked by strong stimuli; it consists of a multineuronal reflex activated by a variety of afferent inputs where the efferent branches involve sympathetic sudomotor fibers. The electrodermal activity reflects sympathetic cholinergic sudomotor function which induces changes in skin resistance to electrical conduction. The response is mediated by ions flux via activation of receptor-coupled Ca^2+^, Cl^−^ and K^+^ channels [24]. Since SSR reflects peripheral C fibers activity, it is considered a reliable quantitative measure of sympathetic function and dysfunction as well as in polyneuropathies and dysautonomic disorders [25–27].

We have previously shown novel serum markers able to indicate cardiovascular diseases, [28, 29] as well as soluble factors able to affect angiogenesis [30] and to discriminate infantile hemangioma from more aggressive vascular malformations or tumors [31]. We also investigated novel molecular markers of melanoma set-up and progression [32].

In the present study, we further addressed the issue of altered angiogenesis in tumor and non-tumor conditions. The expression level of 90 ion-channel genes was investigated in five different solid tumors, having a different histological origin. The expression level of several ion-channels genes was found to be strongly modified; we thus hypothesized that ion-transport may represent a measurable sign of the altered underlying angiogenesis. We, therefore, measured in vivo the ion-channel function in a human vascular malformation model, namely flat port-wine stains, as a model to test electrical-stimuli transport in a human vascular disorder accepted by the Ethic Committee.

## Methods

### Ion-channel gene expression investigation

Gene expression levels were investigated by accessing human cancer datasets available at Oncomine (http://www.oncomine.org). The current Oncomine version contains several hundred different patients-datasets, referring to tumors biopsies obtained from almost any histological source; unfortunately vascular tumors (such as hemangioma, angiomas, hemangioendothelioma, angiosarcoma) are lacking from such database. In the current study 25, independent datasets from histologically different solid tumors were investigated, namely, bladder cancer, glioblastoma, melanoma, breast cancer, lung adenocarcinoma, as indicated in details in Table 1.

**Table 1:** Tumors types, datasets names, patients numerosity and reference of each dataset investigated in the present study, from oncomine database (http://www.oncomine.org)

	Tumor type and dataset name	No of control samples	No of cancer samples	References n.
Superficial bladder cancer				
1	Dyrskjot dataset	14	28	[86]
2	Lee dataset	68	126	[87]
3	Sanchez dataset	48	28	[88]
4	Blaveri dataset	2	25	[89]
Glioblastoma				
5	Lee dataset	3	78	[90]
6	Bredel dataset	4	26	[91]
7	Sun dataset	23	81	[92]
8	Murat dataset	4	80	[93]
Melanoma				
9	Talantov dataset	7	45	[94]
10	Riker dataset	5	14	[95]
11	Critchley dataset	23	23	[96]
12	Haqq dataset	3	23	[97]
Breast invasive ductal cancer				
13	Ma dataset	28	18	[98]
14	Curtis dataset	144	1556	[99]
15	Radvanji dataset	5	26	[100]
16	Turashvi dataset	20	5	[101]
17	Zhao dataset	4	154	[102]
Lung adenocarcinoma				
18	Bhattacharjee dataset	17	139	[103]
19	Beer dataset	10	86	[104]
20	Stearman dataset	19	20	[105]
21	Hou dataset	65	45	[106]
22	Okayama dataset	20	226	[107]
23	Selamat dataset	58	58	[108]
24	Landi dataset	49	58	[109]
25	Su dataset	31	31	[110]
Totals		674	2999	

Table 2 reports the detailed list of the 90 ion-channels and ion-channel related genes investigated in the present study. Briefly, different members were selected from 21 channel families, namely: amiloride-sensitive cation channels, calcium Channels voltage-dependent, cation channels sperm associated, FXYD domain containing ion transport regulators, gamma-aminobutyric acid (GABA) receptors, glutamate receptors ionotropic, potassium channels voltage gated subfamily, cholinergic receptors (Nicotinic), chloride channels, cyclic nucleotide gated channels, glutamate Receptors, sodium leak channels, purinergic receptors P2X, sodium-hydrogen exchanger regulatory factor 4, regulatory solute carrier proteins, sodium channels, glucose activated Ion channels, two pore segment channels, transient receptor potential cation channels, zinc activated ion channels, aquaporins.

**Table 2:** Complete list of ion-channel genes and ion-channel related genes investigated

Gene family	Gene name	Whole gene name
(*A*) *Amiloride-sensitive cation channel*		
1	ACCN1	Amiloride-sensitive cation channel 1
2	ACCN2	Amiloride-sensitive cation channel 2
3	ACCN3	Amiloride-sensitive cation channel 3
4	ACCN4	Amiloride-sensitive cation channel 4
(*B*) *Calcium channel, voltage-dependent*		
5	CACNA1A	Calcium channel, voltage-dependent, P/Q type, alpha 1A Subunit
6	CACNA1B	Calcium channel, voltage-dependent, N type, alpha 1B Subunit
7	CACNA1C	Calcium channel, voltage-dependent, L type, alpha 1C Subunit
8	CACNA1D	Calcium channel, voltage-dependent, L type, alpha 1D Subunit
(*C*) *Cation channel, sperm associated*		
9	CATSPER1	Cation channel, sperm associated 1
10	CATSPER2	Cation channel, sperm associated 2
11	CATSPER3	Cation channel, sperm associated 3
12	CATSPER4	Cation channel, sperm associated 4
(*D*) *FXYD domain containing ion transport regulator*		
13	FXYD1	FXYD domain containing ion transport regulator 1
14	FXYD2	FXYD domain containing ion transport regulator 2
15	FXYD3	FXYD domain containing ion transport regulator 3
16	FXYD4	FXYD domain containing ion transport regulator 4
17	FXYD5	FXYD domain containing ion transport regulator 5
(*E*) *Gamma-aminobutyric acid* (*GABA*) *A receptor*		
18	GABRA1	Gamma-aminobutyric acid (GABA) A receptor, alpha 1
19	GABRB3	Gamma-aminobutyric acid (GABA) A receptor, beta 3
20	GABRP	Gamma-aminobutyric acid (GABA) A receptor, Pi
(*F*) *Glutamate receptor, ionotropic*		
21	GRIA1	Glutamate receptor, ionotropic, AMPA 1
22	GRIN2A	Glutamate receptor, ionotropic, *N*-methyl d-aspartate 2A
23	HTR3A	5-Hydroxytryptamine (serotonin) receptor 3A, ionotropic
24	HTR3B	5-Hydroxytryptamine (serotonin) receptor 3B, ionotropic
(*G*) *Potassium channel, voltage gated subfamily*		
25	KCNE3	Potassium channel, voltage gated subfam.E regulatory beta Sub. 3
26	KCNE4	Potassium channel, voltage gated subfam.E regulatory beta Sub. 4
27	KCNH2	Potassium channel, voltage gated Eag related subfamily H, member 2
28	KCNH1	Potassium channel, voltage gated Eag relat.subfam.H, Member 1
29	KCNJ11	Potassium channel, inwardly rectifying subfamily J, member 11
30	KCNJ12	Potassium channel, inwardly rectifying subfamily J, member 12
31	KCNMA1	Potassium channel, calcium activated large conductance subfam.M alpha, member 1
32	KCNMB2	Potassium channel subfamily M regulatory beta subunit 2
33	KCNMB4	Potassium channel subfamily M regulatory beta subunit 4
34	KCNQ1	Potassium channel, voltage gated KQT-Like Subfam. Q, Member 1
35	KCNRG	Potassium channel regulator, protein CLLD4
36	KCNV1	Potassium channel, voltage gated modifier subfamily V, Member 1
37	KCNN1	Potassium channel, calcium activated intermediate/small conductance subfamily N alpha, member 1
38	KCNN2	Potassium channel, calcium activated intermediate/small conductance subfamily N alpha, member 2
39	KCNN3	Potassium channel, calcium activated intermediate/small conductance subfamily N alpha, member 3
40	KCNN4	Potassium channel, calcium activated intermediate/small conductance subfamily N alpha, member 4
(*H*) *Cholinergic receptor, nicotinic*		
41	CHRNA1	Cholinergic receptor, nicotinic, alpha 1 (muscle)
42	CHRNA2	Cholinergic receptor, nicotinic, alpha 2 (neuronal)
43	CHRNA3	Cholinergic receptor, nicotinic, alpha 3 (neuronal)
44	CHRNA5	Cholinergic receptor, nicotinic, alpha 5 (neuronal)
(*I*) *Chloride channel*		
45	CFTR	Cystic fibrosis transmembrane conductance regulator
46	BEST1	Bestrophin 1
47	CLCN1	Chloride channel 1, skeletal muscle (CIC-1)
48	CLCN5	Chloride channel, voltage-sensitive 5 (CIC-5)
49	CLIC1	Chloride intracellular channel 1
50	CLIC4	Chloride intracellular channel 4
(*L*) *Cyclic nucleotide gated channel*		
51	CNGB3	Cyclic nucleotide gated channel beta 3
(*M*) *Glutamate receptor*		
52	GRID1	Glutamate receptor, ionotropic, delta 1
53	GRID2	Glutamate receptor, ionotropic, delta 2
(*N*) *Sodium leak channel*		
54	NALCN	Sodium leak channel, non selective
(*O*) *Purinergic receptor P2X*		
55	P2RX1	Purinergic receptor P2X, ligand gated ion channel, 1
56	P2RX2	Purinergic receptor P2X, ligand gated ion channel, 2
57	P2RX3	Purinergic receptor P2X, ligand gated ion channel, 3
58	P2RX4	Purinergic receptor P2X, ligand gated ion channel, 4
59	P2RX6	Purinergic receptor P2X, ligand gated ion channel, 6
60	P2RX7	Purinergic receptor P2X, ligand gated ion channel, 7
(*P*) *Sodium-hydrogen exchanger regulatory factor 4*		
61	PDZD3	PDZ domain containing 3
(*Q*) *Regulatory solute carrier protein*		
62	RSC1A1	Regulatory solute carrier protein, family 1, member 1
(*R*) *Sodium channel*		
63	SCN1A	Sodium channel, voltage gated, type I alpha subunit
64	SCN2A	Sodium channel, voltage gated, type II alpha subunit
65	SCN3A	Sodium channel, voltage gated, type III alpha subunit
66	SCN4A	Sodium channel, voltage gated, type IV alpha subunit
67	SCN5A	Sodium channel, voltage gated, type V alpha subunit
68	SCN8A	Sodium channel, voltage gated, type VIII alpha subunit
(*S*) *Glucose activated ion channel*		
69	SLC5A4	Solute carrier family 5 (glucose activated ion channel)
(*T*) *Two pore segment channel*		
70	TPCN1	Two pore segment channel 1
71	TPCN2	Two pore segment channel 2
(*W*) *Transient receptor potential cation channel*		
72	TRPC1	Transient recep. potential cation channel, subfamily C, member 1
73	TRPC2	Transient recep. potential cation channel, subfamily C, member 2
74	TRPC3	Transient recep. potential cation channel, subfamily C, member 3
75	TRPC4	Transient recep. potential cation channel, subfamily C, member 4
76	TRPM1	Transient recep. potential cation channel, subfamily M, member 1
77	TRPM2	Transient recep. potential cation channel, subfamily M, member 2
78	TRPM3	Transient recep. potential cation channel, subfamily M, member 3
79	TRPM4	Transient recep. potential cation channel, subfamily M, member 4
80	TRPM7	Transient recep. potential cation channel, subfamily M, member 7
81	TRPV1	Transient recep. potential cation channel, subfamily V, Member 1
82	TRPV2	Transient recep. potential cation channel, subfamily V, member 2
83	TRPV3	Transient recep. potential cation channel, subfamily V, member 3
84	TRPV4	Transient recep. potential cation channel, subfamily V, member 4
(*Y*) *Zinc activated ion channel*		
85	ZACN	Zinc activated ligand-gated ion channel
(*Z*) *Acquaporins*		
86	AQP1	Aquaporin 1
87	AQP2	Aquaporin 2
88	AQP3	Aquaporin 3
89	AQP4	Aquaporin 4
90	AQP5	Aquaporin 5

Expression of such 90 genes was evaluated in oncomine database by setting “cancer vs. normal analysis” and choosing as cancer type: “superficial bladder cancer” within the bladder cancer group, “glioblastoma” within the brain and CNS cancer group, “breast invasive ductal carcinoma” within the breast cancer group, “lung adenocarcinoma” within the lung cancer group, and “melanoma”.

Expression fold change (cancer vs. normal samples) and *p* values were reported for each analysis. Gene expression in tissue biopsies from 3673 patients was analyzed. Namely, 674 control-samples and 2999 cancer-samples were investigated.

Two additional datasets were identified and investigated within GEO database from NCBI (http://www.ncbi.nlm.nih.gov/gds), namely GSE41614 and GSE44115. Such datasets specifically refer to the vessels component within cancer samples; they were analyzed by means of the GEO2R interface available at the http://www.ncbi.nlm.nih.gov/gds site.

### Patients recruitment

Several reports indicate that direct electrical stimulation may affect cell proliferation and dissemination in oncological setups and may induce other physiological effects [33–39]. Thus a clinical-study involving any electrical stimulation in cancer patients would be not acceptable by the Ethic Committee, according to the articles n. 14 and n. 16 of the Helsinki declaration on biomedical studies involving human patients, and to the article n. 16 of the Oviedo Convention.

For such reasons we submitted to the Ethic Committee the request to authorize the in vivo investigation in non-tumor patients showing a pathological condition resembling, at least in part, the tumor neo-angiogenesis. Such request was authorized by the Ethic Committee and this explains way we investigated the SSR in angioma patients.

Fourteen patients (six males and eight females) affected by cutaneous flat port-wine stains were recruited at IDI-IRCCS, Rome. The study was approved by the institutional review board of IDI-IRCCS Hospital, Rome (IDI Ethic Committee 2011, n. 363). Patients with flat port-wine stains diagnosis (age 18–70 years) undergoing no treatment of any type were consecutively recruited. Patients did not show systemic or neurological disorders nor obvious psychological problems. Physical general and neurological examination were normal in all cases. The average lesion was about 25 cm × 10 cm, typically a portion of the limb. All patients signed an informed consent to participate in the study.

### Sympathetic skin responses SSR recording

SSR study was carried out according to the technical standards of the International Federation of Clinical Neurophysiology [40]. During the test, subjects were kept relaxed with comfortable light and temperature (26–28 °C); the test was started after 5 min of previous adaptation. The apparatus used was an electromyography and evoked potential equipment (MedelecSynergy, Viasys Healthcare, Madison WI USA). Recording electrodes consisted of a pair of superficial electrodes: recording was carried out on the glabrous skin on the flat port-wine stain, and the reference was placed 2 cm away from the lesion. The ground electrode was proximal to the recording electrodes. Electrical stimulation was applied through superficial electrodes over the right median nerve. The stimulus was strong but tolerable (not noxious). The electrical stimulus was applied four times at irregular intervals of 30–60 s (stimulus duration: 0.1 ms; intensity: 80 mA) to avoid habituation, and SSR waves were obtained. SSR recordings were carried out in quadruplicates at the angiomas lesion sites and onto a contralateral healthy skin region in each patient.

The amplifier bandwidth was 0.1–100 Hz. Responses were recorded on the skin with an impedance <5 kΩ. The mean latency and peak-to-peak amplitude were calculated and used for the following analyses.

### Statistical analysis

For gene expression data, the statistically significant threshold originally set at *p* = 0.05 was then corrected according to the Bonferroni correction for multiple comparisons, to take into account the multiple comparisons carried out. Therefore, the final corrected threshold was set at p < 0.0005 (value obtained from 0.05/90 comparisons, for each investigated dataset).

The expression level of any given gene was considered to be significantly altered vs control when the significant *p* value was matched in at least half—or in at least 1—of the specific databases investigated for each tumor type. Namely, regarding the “at least half datasets” stringency level, the significant *p* value had to be matched in at least 2 datasets out of the 4 investigated for bladder cancer; in at least 2 datasets out of the 4 studied for glioblastoma; in at least 2 datasets out of the 4 examined for melanoma; in at least 3 datasets out of the 5 investigated for invasive ductal breast carcinoma; in at least 4 datasets out of the 8 investigated for lung adenocarcinoma.

SSR was recorded four consecutive times for each patient on the diseased skin and four consecutive times on the healthy contralateral skin. Mean ± SE was computed; mean latency and mean peak-to-peak amplitude recorded at the diseased-skin level were compared to measures obtained on the contralateral healthy control site. Paired t Student test was carried out, and statistical significance was set at p < 0.05. Normal distribution of SSR data was tested according to the D’Agostino-Pearson test and carried out by the GraphPad software. Data with normal distribution were analyzed with the paired t test, while data with a not-normal distribution were analyzed with the non-parametric Wilcoxon match-paired signed ranked test.

## Results

### Expression level of ion-channels genes in tumors

The expression level of 90 ion-channels and ion-channels related genes (see Table 2 for the complete list) was analyzed in 3673 human biopsies of 5 histologically different solid tumors, namely: superficial bladder cancer, glioblastoma, melanoma, invasive ductal breast cancer, lung adenocarcinoma, as reported in Table 1 in more details.

Table 3 indicates the ion-channel families showing a significantly altered expression (p < 0.0005). For each cancer type, two columns are presented: the left-hand column (lighten) reports genes indicating a significantly modified expression in at least 1—or less than half-of the investigated datasets. The right-hand column of each tumor type (shadowed) reports in bold all genes showing a significantly modified expression in at least half of the examined datasets (see criteria detailed in “Methods” section). All genes matching the shadowed columns criteria were found overexpressed. More in detail:

**Table 3:**
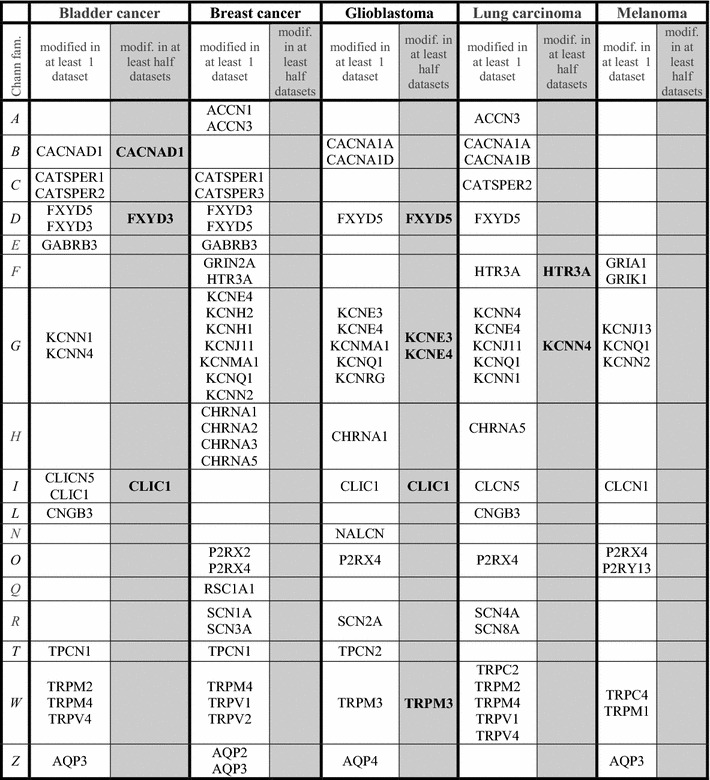
Genes reported show a significantly altered expression in each cancer type

For each cancer type the lighten column reports ion-channels genes significantly modified in at least 1 dataset. The shadowed column reports ion-channels genes significantly modified in at least half of the investigated datasets. Channel family codes as in Table 2

Significance threshold: p < 0.0005 (see “Methods” section)

- Within the calcium channel, voltage-dependent family, expression of CACNAD1 gene was found significantly modified in superficial bladder cancer datasets, namely in Dyrskjot dataset (*p* = 4 × 10^−6^) and in Sanchez dataset (*p* = 5 × 10^−8^), with an average 3.7-fold increase vs. ctrls;
- Within the FXYD domain containing ion transport regulator family, expression of FXYD3 gene was found significantly modified in bladder cancer (Dyskjot dataset, *p* = 1 × 10^−6^, Lee dataset, *p* = 3 × 10^−6^ and Sanchez dataset, *p* = 6 × 10^−21^) with an average 3.3-fold increase vs. ctrls. Furthermore, FXYD5 gene expression was found significantly modified in glioblastoma (in Lee dataset, *p* = 2 × 10^−5^ and in Sun dataset, *p* = 1 × 10^−11^) with an average 2.5-fold increase vs. ctrls;
- Within the glutamate receptor, ionotropic family, expression of HTR3A gene was found significantly modified in 5 lung carcinoma datasets, namely in Beer dataset (*p* = 3 × 10^−8^), in Hou dataset (*p* = 7 × 10^−6^), in Okayama dataset (*p* = 2 × 10^−13^), in Selamat dataset (*p* = 1 × 10^−8^), and in Landi dataset (*p* = 8 × 10^−6^) with an average 5.08 fold increase vs ctrls;
- Within the potassium channel, voltage gated family, expression of three genes was found significantly modified in two cancer types. Namely, KCNE3 and KCNE4 genes are modified in glioblastoma datasets. KCNE3 shows an average 5.3-fold increase vs. ctrls (in Lee dataset, *p* = 3 × 10^−10^ and in Sun dataset, 5 × 10^−9^). KCNE4 shows an average 2.9-fold increase vs ctrls in Lee dataset (*p* = 1 × 10^−8^) and in Sun dataset (*p* = 1 × 10^−13^). KCNN4 was found altered in nearly all lung carcinoma datasets, namely in Bhattacharjee dataset (*p* = 1 × 10^−6^), Stearman dataset (*p* = 3 × 10^−7^), Hou dataset (*p* = 3 × 10^−9^), Okayama dataset (*p* = 1 × 10^−8^), Selamat dataset (*p* = 2 × 10^−14^), Landi dataset (*p* = 2 × 10^−17^) and in Su dataset (*p* = 6 × 10^−8^) with an average 3.6-fold increase vs ctrls;
- Within the chloride channel family, expression of CLIC1 gene was found significantly modified in bladder cancer (in Lee dataset, *p* = 8 × 10^−8^ and Sanchez dataset, *p* = 3 × 10^−5^) with an average 1.5-fold increase vs. ctrls. CLIC1 is also significantly modified in glioblastoma (in Bredel dataset, *p* = 3 × 10^−7^ and Sun dataset, *p* = 2 × 10^−23^) with an average 5.7-fold increase vs. ctrls;
- Within the transient receptor potential cation channel family, expression of TRPM3 gene is altered in glioblastoma, i.e., in the Lee dataset (*p* = 5 × 10^−5^) and in the Sun dataset (*p* = 2 × 10^−5^) with an average 2.3-fold increase vs. ctrls.

Within the shadowed columns, glioblastoma appears to have the highest number of modified ion-channel genes (namely: FXYD5, KCNE3, KCNE4, CLIC1, TRPM3).

Melanoma and breast cancer show no genes in the shadowed columns. However, within the lighten columns, breast invasive ductal cancer and melanoma show significantly modified ion-channel genes from 13 families and 6 families, respectively.

### SSR recording in flat port-wine stains patients

The above reported analyses demonstrated that the expression level of several ion-channels is significantly altered in several human cancer biopsies. Such tumors are histological different. However, they all present an altered vascular tree, due to the tumor neo-angiogenesis. We hence hypothesized that vascular alterations observed in several different cancer types may harbor, at least to a certain extent, the observed ion-channels expression modifications. According to these findings, we hypothesized that measuring ion-channel transport may represent a non-invasive technique to investigate alterations in tumor- as well non-tumor altered angiogenesis. In vivo analysis of ion transport in a vascular malformation, the clinical model was carried out. In fact, measuring in vivo ion-transport in tumor patients was considered ethically not acceptable; we were then forced to identify a non-tumor clinical condition showing clear vascular anomalies. The flat port-wine stains clinical model was approved by the Ethic Committee as a safe model to investigate Sympathetic Skin Responses and electrical signal transport, as the less invasive approach possible in the current study. SSR depends on Ca^2+^, K^+,^ and Cl^−^ channels found to be altered in Table 3. Therefore, 14 patients with flat port-wine stains diagnosis were consecutively recruited, namely eight female (mean age 27 years) and six male (mean age 29.1 years). SSR recordings were carried out in quadruplicates at the angiomas lesion sites and onto a contralateral healthy skin region in each patient. Statistical analysis was performed with paired tests as reported in Methods.

**Fig. 1:**
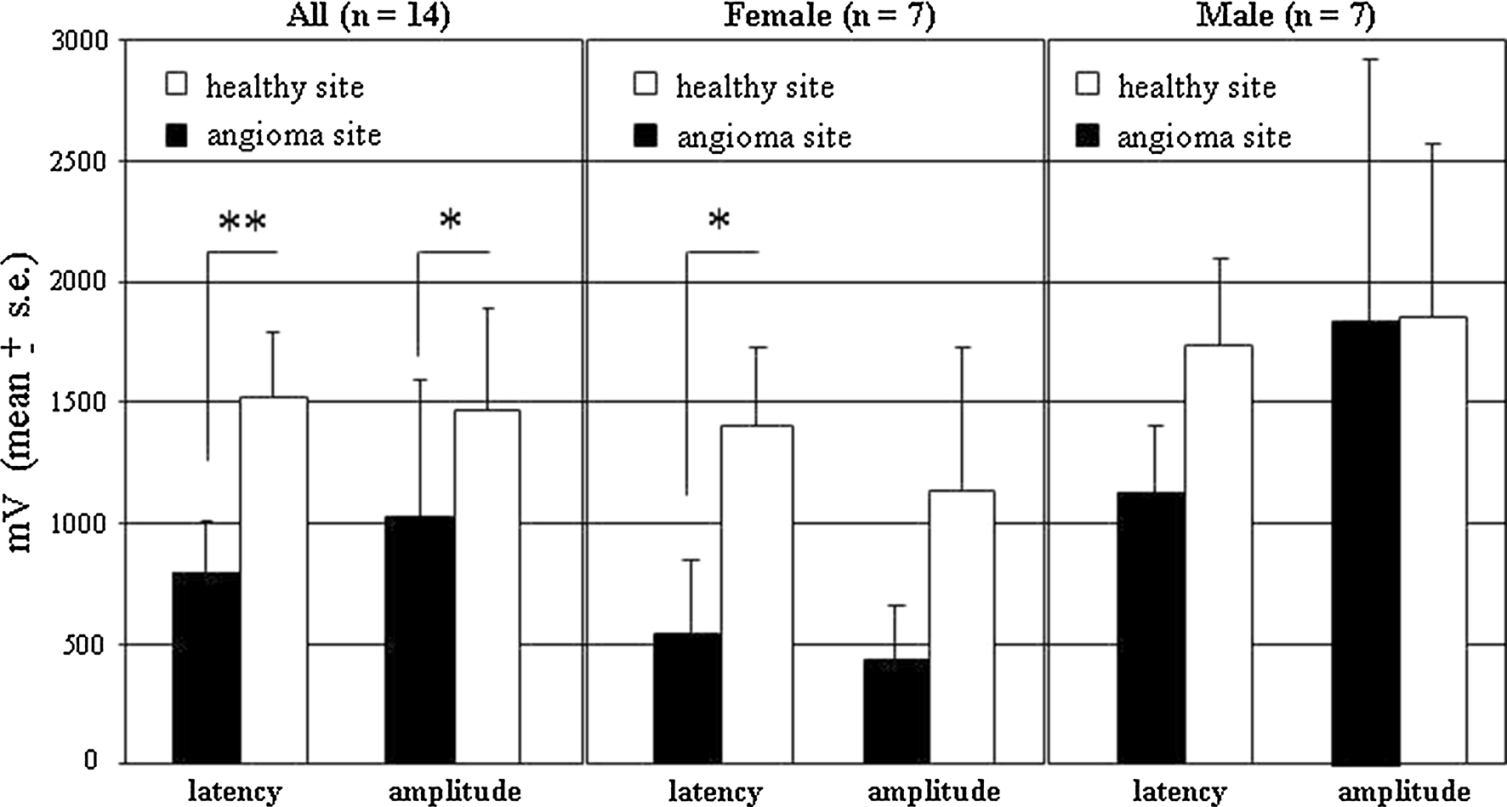
SSR recordings carried out in 14 flat port-wine stains patients. Both latency and amplitude signals were measured on the angioma site and the contralateral healthy site of each patient. A paired test was carried out according to “Methods” section. **indicates p < 0.01; *indicates p < 0.05

Figure 1 shows the mean latency and means amplitude measured at diseased and healthy sites, expressed in mV. A strong and significant reduction in both latency and peak-to-peak amplitude signals was observed in the diseased site as compared to the healthy site, in the whole patients population. When a gender-specific analysis was carried out, both latency and amplitude were strongly reduced in female patients, while latency was reduced in male, although in a not-significant manner.

### Ion channel expression in normal vessels as compared to tumor derived vessels

Data reported in Table 3 and in Fig. 1 led us to hypothesize that ion channels genes may play a relevant role in cancers as well as in other pathological conditions of altered angiogenesis. To further support this hypothesis we investigated two human samples datasets available at GEO database. The first dataset (GSE41614) reports transcriptional profiling of tumor-associated blood vessels in human invasive bladder cancer samples. In such dataset the expression data were obtained on laser capture microdissected vessels isolated from normal bladder tissue or from tumor bladder tissues. Data from ten samples were analyzed, i.e., five normal samples vs five cancer samples.

The second dataset (GSE44115) reports gene expression data in OCT frozen human angiosarcoma compared to OCT frozen normal mesenchymal tissues. In this case 18 angiosarcoma samples were compared to four controls (two from skeletal muscle normal uninvolved tissue and two pooled RNA from normal tissues).

Tables 4 and 5 report the gene name, the log2 fold change, the p values adjusted according to the Benjamini and Hochberg false discovery rate methods, and the rank position (according to the by p value). Several genes, within the top 250 genes most significantly regulated, belong to ion channels families, either in bladder cancer blood vessels (9 genes, Table 4) and in angiosarcoma vessels (123 genes, Table 5). Such data indicate that several ion channels may play a key role in the vessels within cancer tissues.

**Table 4:** Ion channels genes most regulated in bladder cancer vessels vs control vessels

Rank position in the top 250 (by p value)	Gene identifier	Gene name	Adjusted p value*	log2 fold change
16th	KCNC4	Potassium voltage-gated channel subfamily C member 4	0.009	+0.507
42th	KCNG4	Potassium voltage-gated channel modifier subfamily G member 4	0.01	+0.415
75th	VDAC3	Voltage dependent anion channel 3	0.01	−1.511
90th	CRACR2B	Calcium release activated channel regulator 2B	0.01	+0.472
115th	KCNS2	Potassium voltage-gated channel modifier subfamily S member 2	0.01	+0.637
153th	SEC23B	Sec23 homolog B, coat complex II component (involved in vesicle trafficking)	0.01	−0.566
173th	CBARP	CACN Beta subunit associated regulatory protein	0.01	+0.627
218th	P2RX5	Purinergic receptor P2X 5 (ligand-gated ion channel)	0.01	+0.403
247th	SCN2B	Sodium voltage-gated channel beta subunit 2	0.02	+0.487

* According to the Benjamini and Hochberg false discovery rate method

Data from Geo dataset GSE41614, available at http://www.ncbi.nlm.nih.gov/geo/query/acc.cgi?acc=GSE41614)

**Table 5:** Ion channels genes most regulated in angiosarcoma vs controls

Rank position in the top 250 (by value)	Gene identifier	Gene name	Adjusted p value*	log2 fold change
3th	KCNJ16	Potassium voltage-gated channel subfamily J member 16	0.0000003	+6.673
41th	CLCNKA	Chloride voltage-gated channel Ka	0.001	+3.516
52th	HCN2	Hyperpolarization activated cyclic nucleotide gated potassium channel 2	0.001	+2.099
53th	KCNQ2	Potassium voltage-gated channel subfamily Q member 2	0.001	+1.276
61th	FXYD4	FXYD domain containing ion transport regulator 4	0.0009	+6.247
75th	CRACR2B	Calcium release activated channel regulator 2B	0.002	+1.117
104th	AQP10	Aquaporin 10	0.004	+2.096
125th	KCNJ15	Potassium voltage-gated channel subfamily J member 15	0.004	+3.203
147th	KCNK12	Potassium two pore domain channel subfamily K member 12	0.004	+2.008
153th	FXYD2	FXYD domain containing ion transport regulator 2	0.005	+2.596
167th	KCNJ1	Potassium voltage-gated channel subfamily J member 1	0.01	+3.369
250th	AQP2	Aquaporin 2	0.01	+8.359

* According to the Benjamini and Hochberg false discovery rate method

Data from Geo dataset GSE44115, available at http://www.ncbi.nlm.nih.gov/geo/query/acc.cgi?acc=GSE44115)

## Discussion

It is widely known that nerves and vessels follow similar anatomical paths. Often nerves and vessels show an overlapping anatomy with overlapping branches and ramifications. Several molecular factors are reported to control their respective patterns and growth in a coordinated manner [41], including semaphorin, netrin and slit [42], all strongly regulated by Ca^2+^, Na^+^, and Cl^−^ channels [43, 44]. Thus, an architectural or functional modification of the ones may affect the architecture or function of the others. We, therefore, argued that unordered angiogenesis occurring in tumors and vascular malformations may associate to a corresponding unordered nerve formation and therefore to a measurable alteration of the electric stimulus transport. We hypothesized that (i) ion channels (which are known to regulate nerve- and vessel- formation) may show altered expression in tumors and (ii) SSR recording in vascular malformations patients may unreveal a clinical non-invasive sign of the unordered vessels formation.

The expression level of members of several ion-channel families was found significantly modified in histologically different human cancer biopsies, according to the measures reported in Oncomine database, in an almost 4000 patients-vs-controls group. While several genes were found significantly modified (p < 0.0005) in at least one human dataset, we limit here the discussion to the genes reported in the shadowed columns of Table 3 selected according to highly stringent criteria, i.e. to the ion-channel genes found altered in at least half of the investigated databases of each tumor. We report in the current study that such genes show an increased expression in cancer vs. ctrls samples and have in most cases a definite role in vascular biology and/or cancer setup/progression or nervous system biology.

CACNA1D, a calcium related transporter gene, shows an average 3.75-fold increased expression in superficial bladder cancer datasets. No reports are present in literature referring CACNA1D direct link to bladder cancer. Nevertheless, some studies relate its expression- and methylation-level to prostate cancer [45, 46], indicating CACNA1D as a possible regulator of prostate cancer aggressiveness [47], or refer it to CNS disorders [48], diabetes [49] or calcium level within the vessels [50].

FXYD3 shows a 3.3 average fold increase expression in bladder cancer. It encodes a cell membrane protein regulating ion-pumps and ion-channels function and is known to have a role in tumor progression. Its activity is related to glucose and Cl^−^ ions and has been indicated as a possible biomarker in bladder cancer [51, 52] as well as other cancers including breast [53], colorectal cancer [54], endometrial cancer [55] and intrahepatic cholangiocarcinoma [56].

FXYD5 is a transmembrane auxiliary subunit of the Na^+^-K^+^-ATPase; it shows a 2.5-fold increase in glioblastoma. No direct link with glioblastoma has been reported to date, however it has been found up-regulated in adamantinomatous craniopharyngiomas in children [57] and other members of the FXYD family are known to be associated with different cancer types such as urothelial carcinoma [58], esophageal squamous cell carcinoma [59] and cholangiocarcinoma [60]. FXYD channels are involved in the anti-oxidative stress in vascular smooth muscle, thus controlling the vascular tone [61] and blood pressure [62]. Most interestingly, expression of FXYD2 and FXYD4 genes is modified in angiosarcoma vs control human samples (Table 5).

The expression of serotonin receptor HTR3A was found increased by fivefold in lung carcinoma; no direct link has been reported between HTR3A and lung adenocarcinoma, yet; however, nucleotide polymorphisms of this gene have been related to opioid- or nausea/vomiting signaling pathways in cancer patients [63] and to bowel syndrome [64]. HTR3 is the only ligand-gated ion channel among the serotonin receptors, and it has been associated with neurological disorders such as depression [65] or schizophrenia [66]. Most interestingly about the current study, HTR3A acts as a ligand-gated ion channel neurotransmitter, and causes fast, depolarizing responses in neurons (http://www.genecards.org/cgi-bin/carddisp.pl?gene=HTR3A). It is up-regulated in rosacea, i.e., a chronic inflammatory skin disease often showing telangiectasias in the erythematotelangiectatic form (ETR) [67].

Within the potassium intermediate/small conductance calcium-activated channels, expression of 3 genes (namely KCNE3, KCNE4, KCNN4) has been found altered in the current study, namely in glioblastoma and lung adenocarcinoma. While no direct evidence relate KCNE3 or KCNE4 to glioblastoma, KCNE4 is most abundantly expressed in brain [68]; it exerts functions such as controlling the neuronal firing rate, the synaptic transmission [69] and the T-lymphocytes maturation [70] and it is known to regulate K-channels in vascular smooth muscle [71] and more in general neuronal excitability. Furthermore, KCNN4 single-nucleotide polymorphisms have been related to myocardial infarction [72]. Most interestingly, KCNN4 has been linked to vascular cells proliferation [73]. Notably, expression of several potassium channels is modified in bladder cancer vessels vs controls (KCNC4, KCNG4, KCNS2, see Table 4) and in angiosarcoma vs controls (namely KCNJ16, KCNQ2, KCNJ15, KCNJ12, KCNJ1, see Table 5).

Within the Cl- intracellular channels, CLIC1 expression was found altered in bladder cancer and glioblastoma. CLIC1 has been previously found up-regulated in glioblastoma [74], it is involved in different tumors and acts as an oncogene in pancreatic cancer [75], and has been indicated as a possible cancer biomarker [76]. Interestingly, CLIC1 has recently shown a key role in angiogenesis control in combination with integrins [77, 78]. Interestingly, expression of one Cl^−^ channel (namely CLCNKA) is strongly modified in angiosarcoma vs controls (see Table 5).

Within the transient receptor potential cation channels, TRPM3 gene expression was found increased in glioblastoma by 2.3-fold. Its expression has been previously found increased in glioblastoma [79] and is known to exhibit mechanosensitivity contributing to vascular and cardiac functions [80].

SSR is under the direct control of Ca^2+^, K^−^, Cl^−^ ion channels and strictly depends on the sympathetic autonomous nerve function. In the current study, several ion channels related to the Ca^2+^, K^+,^ and Cl^−^ have relevant, and significantly increased expression in different human cancers and SSR was found strongly altered in human vascular malformations. SSR has been previously indicated as a possible useful diagnostic technique in CNS pathologies [81, 82], as well as fibromyalgia [83] and diabetes [84]. Further, autonomic nerve development and function has been recently shown to play a key role in prostate cancer progression [85]. Thus, given the known role of several ion channels in the tumor, nerve, and vascular biology, we hypothesize that the altered SSR observed in vascular skin malformations, and the observed altered ion-channel gene-expression in several tumors may represent phenomena related to the unordered nerve- and vessel formation, common to tumor-related and non-tumor-related vascular anomalies.

SSR recording gave more significant results in female while in male the trend was present but with no statistical significance. Such difference may be related to a different transport of electrical stimuli in the female skin, which appears evident at both healthy (white boxes in Fig. 1) and diseased sites (black boxes in Fig. 1), as compared to male. A gender-related difference in the skin thickness may underlie, at least in part, such observation likely associated with the skin thickness and water retention induced by the menstrual phases and the hormonal status in females.

In conclusion, data reported in the current study allowed us to conclude that ion-channel expression and function may be strongly affected in pathological conditions where vessels’ (and nerves’) architecture is altered. SSR measurement may thus represent a non-invasive useful tool to investigate vascular skin alterations in both tumor and non-tumor conditions.

## Conclusions

The present study reports for the first time a detailed analysis of the expression level of 90 ion-channel genes in 3673 biopsies form humans affected by solid tumors and from healthy controls. At least ten genes from different ion-channels families were found firmly and significantly up-regulated in histologically different tumors having in common the underlying unordered tumor neo-angiogenesis (Table 3). Moreover, expression of at least 20 ion channels has been found to be strongly modified in cancer associated vessels vs controls (Tables 4, 5). The sympathetic skin responses (SSR), an electrical feature closely related to the ion-channels activity, was then measured in vivo and was found strongly modified in the skin of patients affected by the flat port-wine stains vascular malformation. The present study indicates that modified expression and activity of ion channels is likely related to vascular alteration, in both tumor and non-tumor conditions, and suggests the non invasive technique SSR as a simple, useful tool to investigate skin vascular anomalies.
